# A novel approach to understanding outcome selection and reporting during the evaluation of innovative surgery from first in human to pragmatic RCT: an evolution review

**DOI:** 10.1186/1745-6215-16-S2-P162

**Published:** 2015-11-16

**Authors:** Noah Howes, Jelena Savovic, Julian Higgins, Jane Blazeby

**Affiliations:** 1 University of Bristol, Bristol, UK

## 

The evaluation of innovation from early to late phase studies requires appropriate selection and reporting of outcomes. These are well defined for pharmaceutical interventions, but much less well considered for non-pharmaceutical interventions such as surgery. We report a new method - an evolution review - to understand and document the evolution of outcomes reported at each stage of evaluation of novel surgery from first in human to pragmatic multi-centre RCTs.

A search strategy identified all search terms for precursors and derivatives of a novel surgical intervention and was run in Embase and Medline. Abstracts with primary data were included and ordered chronologically, sequentially numbered and then grouped by year. We identified the year of publication of the first-in-human study and used that year to begin the sampling. Three publications were sampled from each year after randomly ordering. An additional sample equal in size to the previous sample was randomly sampled from the remainder. This methodology was chosen as the initial sample ensured representation of each year studied and the second sample added weight to years where there was a higher frequency of studies published.

This new methodology - which we have termed an ‘evolution review’ - aims to use this information to inform guidelines for outcome reporting during the evolution of surgical interventions.

